# Non-conveyance of older adult patients and association with subsequent clinical and adverse events after initial assessment by ambulance clinicians: a cohort analysis

**DOI:** 10.1186/s12873-021-00548-7

**Published:** 2021-12-11

**Authors:** Jakob Lederman, Veronica Lindström, Carina Elmqvist, Caroline Löfvenmark, Gunnar Ljunggren, Therese Djärv

**Affiliations:** 1 Dept. of Clinical Science and Education, Södersjukhuset, Karolinska Institutet, Sjukhusbacken 10, 118 83 Stockholm, Sweden; 2 Academic Emergency Medical Services/AISAB Ambulance care in Greater Stockholm Ltd, Region Stockholm, Sweden; 3 Samariten Ambulance, Stockholm, Sweden; 4 Dept. of Neurobiology, Care Sciences and Society, section of nursing, Karolinska Institutet, Stockholm, Sweden; 5 Dept. of Health and Caring Sciences, Linnaeus University, Växjö, Sweden; 6 Centre of Interprofessional Cooperation within Emergency care (CICE), Linnaeus University, Växjö, Sweden; 7 Dept. of Health promoting science, Sophiahemmet University, Stockholm, Sweden; 8 Division for Family Medicine, Department of Neurobiology, Care Sciences and Society, Karolinska Institutet, Stockholm, Sweden; 9 Academic Primary Health care Centre, Region Stockholm, Stockholm, Sweden; 10 Dept. of Medicine Solna, Karolinska Institutet, Stockholm, Sweden

**Keywords:** Emergency medical services [MeSH], Non-conveyance, Patient safety, Adverse events, Vital signs

## Abstract

**Background:**

Older adults (age ≥ 65 years) represent a significant proportion of all patients who are not transported to hospital after assessment by ambulance clinicians (non-conveyed patients). This study aimed to fill the knowledge gap in the understanding of the prevalence of older adult non-conveyed patients and investigate their characteristics and risk factors for subsequent and adverse events with those of younger non-conveyed patients comparatively.

**Methods:**

This population-based retrospective cohort study included all adult non-conveyed patients who availed the ambulance service of Region Stockholm, Sweden in 2015; they were age-stratified into two groups: 18–64 and ≥ 65 years. Inter-group differences in short-term outcomes (i.e. emergency department visits, hospitalisations, and mortality within 7 days following non-conveyance) were assessed using multivariate regression analyses.

**Results:**

Older adult patients comprised 48% of the 17,809 non-conveyed patients. Dispatch priority levels were generally lower among older non-conveyed patients than among younger patients. Non-conveyance among older patients occurred more often during daytime, and they were more frequently assessed by ambulance clinicians with nonspecific presenting symptoms. Approximately one in five older adults was hospitalised within 7 days following non-conveyance. Patients presenting with infectious symptoms had the highest mortality risk following non-conveyance. Oxygen saturation level < 95% or systolic blood pressure > 160 mmHg had significantly higher associations with hospitalisation within 7 days following non-conveyance in older adult patients.

**Conclusions:**

Older adult patients have an increased risk for adverse events following non-conveyance. In combination with a complex and variating presentation of symptoms and vital signs proved difficult for dispatch operators and ambulance clinicians to identify and assess, the identified risks raise questions on the patient safety of older adult non-conveyed patients. The results indicate a system failure that need to be managed within the ambulance service organisation to achieve higher levels of patient safety for older non-conveyed patients.

**Supplementary Information:**

The online version contains supplementary material available at 10.1186/s12873-021-00548-7.

## Background

By its very nature, the ambulance service constitutes a high-risk patient safety environment which provides round-the-clock, year-round care services to a large volume of patients with a wide range of medical complaints [1]. Patients who are not transported to the hospital after an initial assessment by ambulance clinicians, that is, non-conveyed patients in the ambulance service [2], represent a significant and increasing proportion of those who seek ambulance services [3–6]. The research on patient outcome following non-conveyance is relatively sparse and, to some extent, has contrasting results [7–9]. However, in the general population, non-conveyance is associated with an increased risk for subsequent and adverse events, such as visits to the emergency department (ED), hospital admissions and even death [8]. In contrast, in another study, the 30-day mortality rate was lower among non-conveyed patients, when compared with all conveyed patients [9]. Therefore, the comparisons between previously published research results should be cautiously undertaken due to the differences in research methodologies, such as the absence of consensus on the relevant outcome measures following non-conveyance. Nonetheless, the differences related to the specific characteristics of ambulance services are to be considered, including the use of non-conveyance guidelines and differences in the formal competence of the ambulance clinicians [10]. The general non-conveyance population are younger than the conveyed patients [3, 7]; however, even within the relatively large group of non-conveyed patients, a significant proportion comprises patients older than 65 years. This group of older adult patients who have not been conveyed are described as a vulnerable group due to their characteristics of complex comorbidity, polypharmacy, and different symptom presentation [11, 12]. A few studies have been conducted with a focus on older adult non-conveyed patients for specific complaints, such as falls [13–15]. Approximately 60% of the general non-conveyance population have been shown to present at least one abnormal vital sign [16]; however, to our knowledge, there is no published study which investigated the potential associations between abnormal vital signs and short-term outcomes among older adult non-conveyed patients.

Therefore, with an aim to increase the understanding of elderly non-conveyed patients, the primary objective of this study was to present the prevalence of older adult non-conveyed patients and their characteristics and, in comparison with younger non-conveyed patients, identify and describe the risk factors associated with ED visits, hospitalisations and mortality up to 7 days following non-conveyance. The secondary objective of this study was to investigate the probable associations between abnormal vital signs and ED visits, hospitalisations and mortality up to 7 days after non-conveyance among older adult non-conveyed patients.

## Methods

### Study design

This population-based retrospective cohort study was conducted in Region Stockholm, Sweden, with the approval of the Regional Ethical Review Board of Stockholm (2017/2187–31) and complied with the guidelines specified by the Strengthening The Reporting of Observational Studies in Epidemiology (STROBE) statement [17]. The definition of non-conveyance within the ambulance service which is used by the National Health Service in England, that is, “a term used to describe a 999 call to the ambulance service which results in a decision not to transport the patient to a health-care facility”, was applied in this study [2]. Only patients that were seen by ambulance clinicians and then discharged at scene was defined as non-conveyed and thus included in this study.

### Setting

All non-conveyed patients who were cared for by the ambulance service of Region Stockholm were included retrospectively over a 1-year time period from 1 January 2015 to 31 December 2015. The study area had a population of approximately 2.2 million inhabitants [18]. The ambulance service is a publicly funded health care service, and approximately 210,000 ambulance assignments are performed annually, of which 14% ends in non-conveyance [19]. The ambulance service can be reached through the national emergency number (112), and all ambulance assignments are dispatched by the regional emergency medical communication centre. Using a symptom-based clinical decision support system called the Swedish Medical Index, the dispatch operator decides whether an ambulance should be dispatched [20]. During the study period, each dispatched ambulance assignment was prioritised on a three-level scale, with the highest level set as Priority 1, followed by Priority 2 and 3. The national regulations stipulate that at least one of the two ambulance clinicians should be a registered nurse. Moreover, the regional regulations stipulate that at least one of the two ambulance clinicians should have undergone a 1-year additional university training and hold a degree in specialist nursing [21]. The medical responsibility within the ambulance team is vested with the specialist nurse [22]. However, specific non-conveyance training has been shown to be missing in the specialist nurse training program [23]. The clinical assessments conducted by the ambulance clinicians includes the usage of a triage tool called the Rapid Emergency Triage and Treatment System (RETTS©) [24]. The patients’ vital signs and chief complaints constitute the basis of the RETTS assessment, and one out of four different priority levels (red, orange, yellow and green) should be decided upon, with red indicating the highest and green the lowest priority. Level Green indicates that the vital signs are unaffected and, most often, the absence of signs of disease as well [24]. The regional guideline stipulates that patients suitable for non-conveyance should be prioritised by the specifications for Level Green in RETTS [25].

### Data collection

**Fig. 1:**
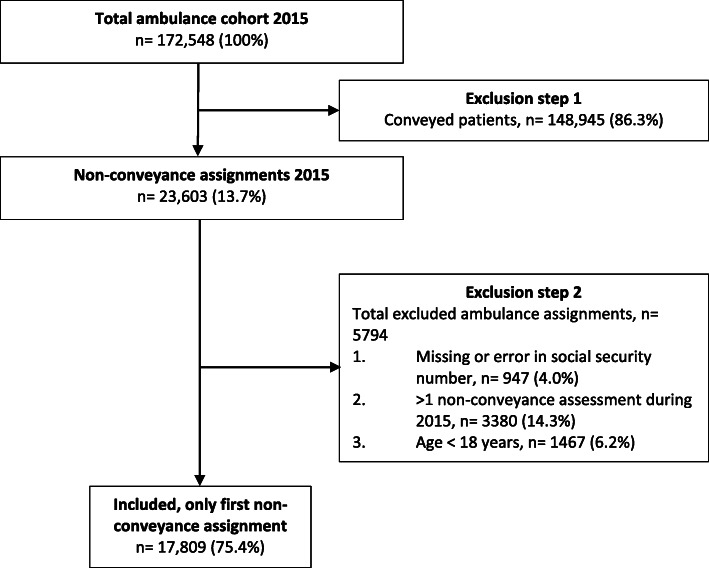
Flow chart over included and excluded non-conveyance assignments in Stockholm, Sweden, 2015

The inclusion criteria were: (1) ambulance assignment ending in non-conveyance; (2) patient age > 18 years. The exclusion criterion was: (1) missing or incomplete social security number (Fig. 1). For all of the study participants, only the first registered non-conveyance event during 2015 was considered. The short-term outcome data were successfully linked through The Regional Health Care Data Warehouse (VAL) by the ambulance assignment numbers. The VAL compiles and stores data on regional health-care use, which ranges from out-patient care (e.g. primary care, ambulance care, and specialist open care) to in-patient care [26]. After successful linkage, the final dataset included data from the electronic ambulance medical records (CAK-net; Region Stockholm) and VAL. The Regional Ethical Review Board of Stockholm waived the need for informed consent. The final dataset was fully anonymized before analysis.

### Study outcomes

Primary endpoints investigated was short-term outcomes (subsequent events); ED visits, hospitalisations and mortality, all within 7 days following non-conveyance. Each single end-point outcome was stratified into three subgroups: 0–1 day, 2–3 days and 4–7 days. The binary variables for each short-term outcome (ED visits, hospitalisations and mortality), which included all events that occurred within a 7-day period, were created to investigate risk factors. Possible associations between hospitalisation and abnormal vital signs was used as secondary endpoint, the binary variable ‘hospitalisation within 7 days following non-conveyance’ was used as the dependent variable and, therefore, was considered an adverse event.

### Data analysis

Age was stratified into a dichotomous variable (younger patients, 18–64 years; older adult patients, ≥ 65 years) and further categorised as a variable which comprised five age levels with 10-year intervals (i.e., 18–64, 65–74, 75–84, 85–94 and ≥ 95 years). Based on the different availability of primary care units and minor EDs in the region of Stockholm during the study period, three-time intervals were generated (day, 8:00 am to 4:00 pm; evening, 4:01 to 10:00 pm; and night, 10:01 pm to 7:59 am). Moreover, four geographical values were created (highly urban, urban, average urban and rural) [27]. The National Advisory Committee for Aeronautics score [28], wherein higher values indicate a more severe condition, was modified as a seven-level scale by excluding the final eighth level (deceased patients). The ambulance medical records were to obligatorily include at least one prehospital initial assessment code which is registered by the ambulance clinician, based on a categorisation of medical conditions by the signs and symptoms. Originally, the dataset comprised 140 different prehospital assessment codes, which were aggregated into 10 categories. Validity during the categorisation process was maintained through the use of the regional medical guidelines for the overall categorisation of signs and symptoms [22]. This process was undertaken by the first author with support from two co-authors. Different cut-off points for abnormal vital signs were defined on the basis of the RETTS (Additional file 1).

### Statistical analysis

Descriptive statistics are presented as percentages, interquartile range (IQR) or median where applicable. The inter-group differences between the younger and older adult non-conveyed patient groups (18–64 years and ≥ 65 years, respectively) were calculated with the chi-square tests (Spearman’s rank statistics were used for continuous variables, and the Kruskall–Wallis test was used for dichotomous variables), Cramer’s V-tests and Student’s *t*-tests where applicable, with the significance level set at 0.05. All tests were two sided. Multivariable logistic regressions analysis was conducted for analysing all short-term outcomes. The goodness of fit for each final model was evaluated using the Hosmer–Lemeshow test and the area under the curve. The crude (COR) and adjusted (AOR) odds ratios with 95% confidence intervals (CI) were calculated. All statistical analyses were conducted by using STATA version 15.1 (StataCorp. 2017; *Stata Statistical Software: Release 15*. College Station, TX: StataCorp LLC).

## Results

The final study population of eligible non-conveyed patients during 2015 in Region Stockholm, Sweden, comprised 17,809 patients. The number of the excluded non-conveyance assignments was 5794 (Fig. 1). The cohort consisted of two relatively even groups of patients (older adult patients 48%; Table 1). The median age among the older adult and younger patients was 85 and 40 years (IQR: 75–94 and 29–52; Table 1), respectively. Overall, older adult patients were more often female than younger patients (54 and 51%, respectively; *p* < 0.001; Table 1), and the dispatch priority was generally lower among the older patients (Table 1). There was a higher prevalence of non-conveyance assessments during the daytime, and lower during the nights, among older patients (*p* < 0.001; Table 1). The majority of patients in both groups were assessed as the lowest RETTS-triage level (*p* = 0.002; Table 1). Furthermore, for both groups, all 10 possible prehospital initial assessment codes were present. However, older adult patients were more often classified as having nonspecific complaints ‘Other/Non-classifiable symptoms’ (41 and 31%, respectively; *p* < 0.001; Table 1) and less frequently classified as ‘Trauma’ (5 and 11%, respectively; *p* < 0.001; Table 1). Older adults commonly presented with at least one abnormal vital sign (35 and 23%, respectively; *p* < 0.001; Table 1).

**Table 1:** Baseline characteristics among non-conveyed patients 18–64 years and > 65 years in Stockholm, Sweden, 2015

Variable	Age 18–64 ***n*** = 9332	Age ≥ 65 ***n*** = 8476	***p***-value^***a***^
**Sex n (%)**			
Male	4596 (49.2)	3853 (45.5)	< 0.001
Female	4736 (50.8)	4623 (54.5)	
**Age years median (IQR)**	40.4 (29–52)	84.9 (75–94)	< 0.001^b^
**Dispatch priority n (%)**			
Priority 1	5426 (58.2)	3258 (38.5)	< 0.001
Priority 2	3597 (38.5)	4494 (53.0)	
Priority 3	309 (3.3)	721 (8.5)	
**Time of day n (%)**			
Day (8 am-4 pm)	2781 (30.2)	3571 (42.4)	< 0.001
Evening (4 pm–10 pm)	3013 (32.7)	2603 (30.9)	
Night (10 pm-8 am)	3428 (37.2)	2255 (26.7)	
**Geographical location n (%)**			
Highly urban	2748 (29.8)	2195 (26.0)	< 0.001
Urban	5847 (63.4)	5561 (66.0)	
Average urban	482 (5.2)	426 (5.1)	
Rural	145 (1.6)	247 (2.9)	
NACA score* (±SD)	1.48 (±0.96)	1.43 (±0.97)	< 0.001^b^
**On-scene triage level n (%)**			0.002
Triage 1 (highest level)	49 (0.7)	85 (1.2)	
Triage 2	470 (6.6)	402 (5.9)	
Triage 3	1647 (23.3)	1568 (23.0)	
Triage 4 (lowest level)	4917 (69.4)	4770 (69.9)	
**Prehospital initial assessment code**			
Other/Non-classifiable symptoms	2851 (30.6)	3459 (40.8)	< 0.001
Nervous symptoms	1792 (19.2)	1097 (12.9)	
Circulatory symptoms	737 (7.9)	876 (10.3)	
Digestive and abdominal symptoms	785 (8.4)	690 (8.1)	
Respiratory symptoms	722 (7.7)	675 (8.0)	
Medical symptoms	404 (4.3)	582 (6.9)	
Psychiatric symptoms	780 (8.4)	553 (6.5)	
Trauma	1038 (11.1)	383 (4.5)	
Infectious symptoms	146 (1.6)	159 (1.9)	
Obstetrics and gynaecologic symptoms	77 (0.8)	3 (0.04)	
**At least one abnormal vital sign**			
Yes	1888 (23.2)	2724 (34.5)	< 0.001
No	6251 (76.8)	5168 (65.5)	

^*a*^*X*^2^-test, ^b^ = T-test

*NACA-score: The National Advisory Committee for Aeronautics (NACA) score, higher values indicate a more severe condition

### Short-term outcomes and risk factors

In both groups, the highest incidence among all three short-term outcomes occurred within 1 day (Table 2). Except for ED visits within 1 day, older adults visited the ED to a greater extent than younger patients, both at 2–3 and 4–7 days (AOR: 1.45; 95% CI 1.25–1.68 and 1.51; 95% CI 1.24–1.84, respectively; Table 2), following non-conveyance. Approximately one in five of the older adults was subsequently hospitalised within 7 days compared to one in eight among the younger non-conveyed patients. Mortality ratios following non-conveyance were higher in the older adult population, with the highest odds occurring within 1 day (AOR: 13.24; 95% CI 3.03–57.88; Table 2). The following factors were consistently associated with a significantly higher likelihood of all measured subsequent events: The National Advisory Committee for Aeronautics score, highest RETTS triage level, and the prehospital initial assessment code ‘medical symptoms’ (Table 3). The prehospital assessment code ‘Infectious symptoms’ had the highest odds of mortality among older adult patients (AOR: 9.80; 95% CI 2.02–47.85; Table 3). It is noteworthy that there was a relatively high likelihood of mortality among older adults with psychiatric symptoms (AOR: 4.19; 95% CI 6.01–16.61; Table 3). Dispatch priority level 3, assignment during daytime, prehospital initial assessment code ‘other/non-classifiable symptoms’, and having at least one abnormal vital sign were all associated with a significantly higher likelihood of subsequent ED visits and hospitalisations, but not with mortality, following non-conveyance (Table 3).

**Table 2:** Distribution of and adjusted odds-ratios for short-term outcomes among non-conveyed patients in Stockholm, Sweden, 2015

Short-term outcome	Age 18–649332	Age ≥ 658477	COR (95% CI)^**a**^	AOR (95% CI)^**a**^
**ED visit (n %)**				
Within 1 day	1610 (17.25)	1365 (16.10)	0.92 (0.85–0.99)	0.98 (0.89–1.09)
Between 2 and 3 days	450 (4.82)	674 (7.95)	1.70 (1.51–1.93)	1.45 (1.25–1.68)
Between 4 and 7 days	268 (2.87)	369 (4.35)	1.54 (1.31–1.81)	1.51 (1.24–1.84)
**Hospitalisation (n %)**				
Within 1 day	728 (7.80)	808 (9.53)	1.25 (1.21–1.38)	1.41 (1.23–1.61)
Between 2 and 3 days	302 (3.24)	600 (7.08)	2.28 (1.98–2.62)	2.14 (1.79–2.56)
Between 4 and 7 days	170 (1.82)	408 (4.81)	2.73 (2.73–3.27)	2.59 (2.08–3.24)
**Mortality (n %)**				
Within 1 day	4 (0.04)	35 (0.41)	9.67 (3.43–27.21)	13.24 (3.03–57.88)
Between 2 and 3 days	5 (0.05)	29 (0.34)	6.40 (2.48–16.55)	7.20 (2.10–24.64)
Between 4 and 7 days	3 (0.03)	34 (0.40)	12.52 (3.84–40.79)	7.44 (2.16–25.58)

^a^Younger non-conveyed patients, 18–64 years, as reference group.Adjusted for gender, dispatch priority, time of day, geographical location, NACA-score, prehospital initial assessment code, at least one abnormal vital sign and triage level

**Table 3:** Summary of logistic regression models with odds-ratios of risk factors associated to short-term outcomes (within 7-days) among older adult non-conveyed patients in Stockholm, Sweden, 2015

Variable	ED visit		Hospitalisation		Mortality	
	COR (95% CI)	AOR (95% CI)	COR (95% CI)	AOR (95% CI)	COR (95% CI)	AOR (95% CI)
Gender						
Male	1	1	1	1	1	1
Female	1.15 (1.04–1.28)	1.09 (0.96–1.25)	1.13 (1.02–1.25)	1.05 (0.93–1.19)	1.20 (0.80–1.79)	1.38 (0.84–2.28)
Dispatch priority						
Priority 1	0.41 (0.37–0.46)	0.46 (0.40–0.53)	0.41 (0.37–0.46)	0.44 (0.38–0.50)	0.42 (0.27–0.64)	0.42 (0.24–0.73)
Priority 2	1	1	1	1	1	1
Priority 3	2.28 (1.88–2.77)	2.04 (1.61–2.57)	2.57 (2.13–3.10)	2.27 (1.81–2.85)	1.05 (0.48–2.31)	0.35 (0.08–1.45)
Time of day						
Day (8 am-4 pm)	1.71 (1.51–1.94)	1.72 (1.47–2.01)	1.66 (1.47 (1.87)	1.64 (1.41–1.89)	0.98 (0.61–1.59)	0.83 (0.47–1.49)
Evening (4 pm–10 pm)	1	1	1	1	1	1
Night (10 pm-8 am)	0.68 (0.59–0.79)	0.69 (0.58–0.83)	0.65 (0.57–0.75)	0.63 (0.53–0.74)	0.79 (0.48–1.30)	0.63 (0.34–1.18)
Geographical location						
Highly urban	0.84 (0.74–0.95)	0.93 (0.80–1.08)	0.83 (0.74–0.93)	0.90 (0.78–1.04)	1.10 (0.70–1.73)	1.39 (0.80–2.41)
Urban	1	1	1	1	1	1
Average urban	0.95 (0.75–1.21)	0.95 (0.71–1.27)	0.91 (0.72–1.15)	0.91 (0.68–1.19)	1.97 (0.97–4.01)	3.63 (1.69–7.79)
Rural	2.07 (1.50–2.85)	1.78 (1.14–2.76)	1.88 (1.37–2.56)	1.50 (0.97–2.33)	2.52 (0.91–7.02)	0.43 (0.05–3.56)
NACA-score*	1.29 (1.23–1.36)	1.22 (1.13–1.33)	1.26 (1.20–1.33)	1.20 (1.11–1.29)	1.85 (1.54–2.22)	1.48 (1.11–1.97)
On-scene triage level						
Triage 1 (highest level)	3.24 (2.05–5.10)	1.98 (1.19–3.30)	3.39 (2.17–5.31)	2.33 (1.41–3.84)	9.69 (3.79–24.78)	6.30 (2.12–18.64)
Triage 2	1	1	1	1	1	1
Triage 3	0.76 (0.60–0.96)	0.77 (0.59–1.01)	0.76 (0.61–0.95)	0.81 (0.62–1.05)	0.75 (0.33–1.71)	0.65 (0.27–1.58)
Triage 4 (lowest level)	0.66 (0.53–0.82)	0.73 (0.56–0.95)	0.67 (0.54–0.82)	0.75 (0.58–0.97)	0.41 (0.19–0.89)	0.46 (0.19–1.13)
Prehospital initial assessment code						
Circulatory symptoms	1	1	1	1	1	1
Nervous symptoms	0.52 (0.41–0.67)	0.53 (0.40–0.71)	0.53 (0.42–0.67)	0.59 (0.45–0.77)	1.00 (0.31–3.27)	1.61 (0.39–2.41)
Digestive and abdominal symptoms	1.26 (0.99–1.61)	1.22 (0.92–1.62)	1.21 (0.96–1.54)	1.21 (0.92–1.59)	4.35 (1.47–12.84)	7.86 (2.10–29.39)
Infectious symptoms	1.69 (1.16–2.47)	1.21 (0.77–1.90)	1.77 (1.24–2.54)	1.34 (0.87–2.08)	4.53 (1.12–18.28)	9.80 (2.02–47.45)
Medical symptoms	1.60 (1.22–2.10)	1.66 (1.20–2.30)	1.67 (1.28–2.16)	1.94 (1.42–2.65)	3.73 (1.14–12.18)	8.01 (1.91–33.67)
Obstetrics and gynaecologic symptoms	Empty	Empty	Empty	Empty	Empty	Empty
Other/Non-classifiable symptoms	1.15 (0.94–1.41)	1.45 (1.14–1.84)	1.22 (1.01–1.48)	1.59 (1.26–2.00)	1.75 (0.61–4.99)	3.39 (0.91–12.59)
Psychiatric symptoms	0.78 (0.60–1.02)	0.81 (0.59–1.10)	0.77 (0.59–1.00)	0.83 (0.61–1.12)	3.23 (1.05–9.94)	4.19 (1.06–16.61)
Respiratory symptoms	0.84 (0.64–1.11)	1.02 (0.74–1.41)	0.82 (0.63–1.06)	1.03 (0.76–1.41)	1.58 (0.44–5.63)	1.39 (0.22–8.81)
Trauma	0.53 (0.40–0.70)	0.49 (0.35–0.69)	0.58 (0.45–0.75)	0.54 (0.39–0.74)	0.96 (0.26–3.57)	2.39 (0.53–10.69)
At least one abnormal vital sign						
Yes	2.08 (1.85–2.34)	2.25 (1.19–3.30)	2.10 (1.88–2.35)	2.35 (2.04–2.71)	3.04 (2.01–4.61)	1.63 (0.92–2.89)
No	1	1	1	1	1	1

Younger non-conveyed patients, 18–64 years, as reference group

Short-term outcomes include all events within respective short-term outcome occurring within 7-days following non-conveyance

COR = Crude odds-ratio, AOR = Adjusted odds-ratio

*NACA-score: The National Advisory Committee for Aeronautics (NACA) score, higher values indicate a more severe condition

### Abnormal vital signs association with hospitalisation

**Fig. 2:**
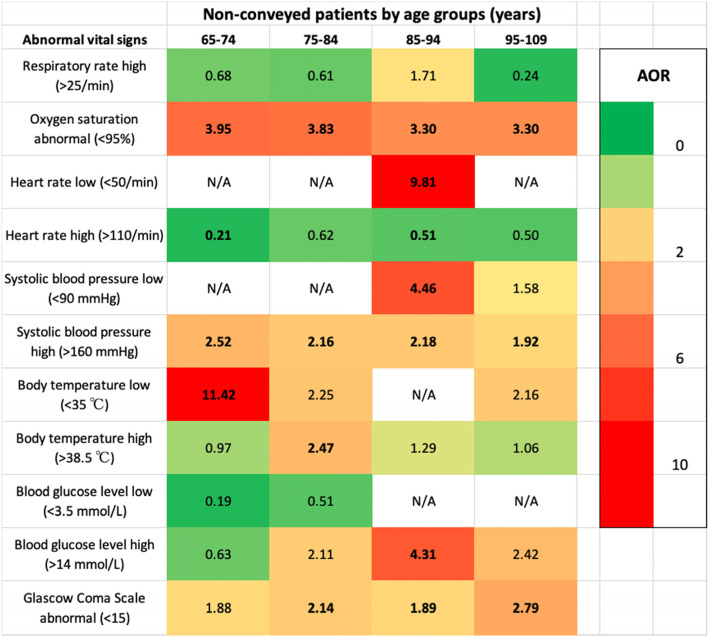
Heatmap illustrating adjusted odds-ratios for abnormal vital signs association to hospitalisation within 7 days after non-conveyance in Stockholm, Sweden, 2015. Younger non-conveyed patients, 18–64 years, as reference group. Bold text = CI below/above 1, i.e. significant. ‘Respiratory rate low’ excluded because of no events. N/A = No cases. All included analyses were adjusted for gender, dispatch priority, time of day, geographical location, NACA-score, prehospital initial assessment code and triage level. AOR = Adjusted odds-ratio. See Additional file 2 for COR and 95% CI

The association between abnormal vital signs and hospitalisation within 7 days following non-conveyance among older adult patients is illustrated in a heat map (Fig. 2). The regression analyses showed a range of associations between abnormal vital signs and hospitalisation among the stratified age-groups (10-year interval). Two categories of abnormal vital signs were associated with a significantly higher likelihood of hospitalisation across all four subgroups: oxygen saturation level < 95% and systolic blood pressure > 160 mmHg. Older adults in the age range of 75–84 years presenting with low oxygen saturation levels at the initial assessment had a seven-folded higher likelihood of hospitalisation within 7 days following non-conveyance than younger patients (AOR: 7.00; 95% CI 4.36–11.25; Fig. 2). A Glasgow Coma Scale (GCS) < 15, which indicates impaired consciousness, demonstrated a significantly increased likelihood for hospitalisation within 7 days following non-conveyance in three out of four subgroups of older adult patients. Patients older than 95 years with an abnormal GCS had the highest likelihood of hospitalisation (AOR: 2.79; 95% CI 1.72–4.52; Fig. 2). Older adult patients in the age group of 65–74 years with a body temperature below 35 °C had an 11 times higher likelihood of hospitalisation than younger patients (AOR: 11.42; 95% CI 2.56–51.03; Fig. 2). Among all subgroups of older adult patients, a heart rate > 110/min decreased the likelihood of hospitalisation (Fig. 2).

## Discussion

Older adult non-conveyed patients (≥65 years) have significantly different clinical characteristics and attributes than younger patients (18–64 years). The findings indicate that the dispatch priority levels are generally lower among older adults. There is a greater incidence of non-conveyance during daytime, and older adult non-conveyed patients are more often assessed by ambulance clinicians to have nonspecific complaints and less often symptoms related to trauma. Despite the lower dispatch levels and symptoms categorised as nonspecific, all measured short-term outcomes over a 7-day period following non-conveyance were more common among older adult patients. Approximately one in five older adult non-conveyed patients was hospitalised within 7 days following non-conveyance. Overall, the increased risk of hospitalisation and mortality following non-conveyance among older adult patients is important new knowledge which raises questions pertinent to patient safety.

This study is one of very few studies to have investigated the general older adult non-conveyance population and to have comparatively evaluated the short-term outcomes with those in younger non-conveyed patients. One of the most important findings from this study is the higher risk of subsequent and adverse events among older non-conveyed patients. The overrepresentation of older adult patients among the lower dispatch levels in combination with an overall higher risk of subsequent events following non-conveyance indicates a complexity which accompanies both symptom presentation and communication in the older adult patient population. These findings may be an effect of age, though age is unaccounted for in the regional medical non-conveyance guideline and the triage system which is currently in use. Older adult non-conveyed patients were more often assessed with nonspecific or vague presenting symptoms than younger patients. In line with a previous study [3], patients assessed with nonspecific presenting symptoms were at higher risk of ED visits and hospitalisation following non-conveyance. Conditions related to infectious diseases, such as sepsis, have high mortality rates among older adult patients [29]. However, we cannot conclude from the results of this study as to which findings in older patients presenting with infectious symptoms conferred the highest mortality risk following non-conveyance, though the missed identification of sepsis could be a possible explanation to our findings. Furthermore, the increased risk of adverse events among older patients with psychiatric symptoms calls for further studies which investigate this vulnerable group of patients.

Furthermore, similarly as in a previous study [8], the occurrence of at least one abnormal vital sign was associated with an increased likelihood of an ED visit and hospitalisation, but not mortality. Renewed contact with the ambulance service and ED visits among older adult non-conveyed patients is relatively common [30], although comparisons with earlier non-conveyance studies are difficult to perform due to significant heterogeneity between these studies [10]. The follow-up time varies greatly between earlier non-conveyance studies: the longer the follow-up time, the greater is the risk that the patient is likely to be affected by other factors than the one which was initially associated with the non-conveyance assessment [7, 8]. There is an absence of consensus with regard to relevant outcome measures for non-conveyed patients, and those must be selected appropriately to capture the specific needs of non-conveyed patients. As shown in this study, patient risk factors had a variable strength of association with the different short-term outcomes which were studied. This implies the need for future research on relevant outcome measures for non-conveyed patients, and what is to be considered as an adverse event in a specific non-conveyance context.

To our knowledge, this is the first study to investigate the association of abnormal vital signs with short-term outcomes among older adult non-conveyed patients. Marked variation was noted among the different age groups of older adult non-conveyed patients. However, the validity of abnormal vital signs in recognising deterioration among older patients has been questioned earlier. The identification of clinical status deterioration among older patients most frequently include other indicators, such as comorbidities, polypharmacy, and behavioural changes and everyday capabilities [31]. The abovementioned findings imply the need for further studies to enhance the knowledge of the potential clinical significance of abnormal vital signs and other indicators in the non-conveyance context. An oxygen saturation level < 95% and systolic blood pressure > 160 mmHg had a significantly higher association with adverse outcomes among all groups of older adult non-conveyed patients in this study. An elevated systolic blood pressure among older adult patients is a relatively common clinical finding and is associated with an increased risk for the development of several different diseases (e.g. coronary heart disease and cerebrovascular disease) [32]. Nonetheless, the results of this study are exploratory in nature and, thus, we cannot definitively conclude the clinical significance of the abnormal oxygen level and elevated systolic blood pressure. Cognitive impairment among older adult patients who visit the ED is associated with adverse outcomes [33]. A GCS score < 15 was associated with adverse outcomes in three out of four groups of older non-conveyed patients in this study.

### Limitations and strengths of this study

This study has limitations which affect its generalisability and validity. Though the study setting included the whole region’s ambulance service, the study should be considered as a single-region study; therefore, the results might not be applicable to other national or international ambulance services and non-conveyance populations. The use of medical records as the data source is associated with methodological challenges, as they were not designed for research purposes, thereby creating unfavourable circumstances from a research perspective. For example, the medical records lacked information on possible patient referral, such as referral to own transportation to ED (e.g. patient’s own car). Moreover, decreasing the risk of outcome bias through misclassification of exposure resulted in the exclusion of ED visits as a possible adverse event. We therefore believe that (unplanned) hospitalisations within 7 days more accurately reflect possible patient safety risks following non-conveyance. Nonetheless, the associations identified in the multiple regression analyses should be considered preliminary. Other studies have identified comorbidities as a risk factor for adverse events [34, 35]. Unfortunately, our data set did not contain information on the patient’s comorbidities. Furthermore, the data set is from 2015 and, therefore, even if the standard of the regional non-conveyance medical guideline, ambulance clinicians’ formal competence, and education has not changed substantially, the results cannot account for any changes which may have influenced the short-term patient outcomes since 2015. When investigating patient risk factors, the short-term outcome mortality was excluded as a dependent variable from our regression models due to the very few events in our data set. Moreover, using mortality as a short-term outcome requires several additional measures to minimise the risk of misclassification of exposure, and includes trying to connect the cause of death with the reason for non-conveyance by including the death certificate in the analysis, which we could not access in this study. The same reasoning applies for hospitalisation. We did not have information on the reasons for hospitalisations, which could be considered a limitation of this study.

Notwithstanding these limitations, this study has several strengths. Our data set comprised all non-conveyed patients in the study setting area during 2015, thereby reducing the possible selection bias. The use of the regional database VAL as a validated outcome register is associated with several methodological advantages [26]. The risk which accompanies the database linkage was minimised through the comprehensive content of VAL with regard to in- and outpatient care in Region Stockholm. Furthermore, the patient’s anonymity was maintained through the database linkage with the use of ambulance assignment numbers. This was one of very few published non-conveyance studies in which the association of abnormal vital signs with short-term outcomes among older adult non-conveyed patients was investigated. This was made possible through the use of ambulance medical records wherein the vital signs of non-conveyed patients were documented.

### Implications for clinical practice and research

The results of this study have important implications for clinical practice and future research. Ambulance stakeholders, health-care policymakers, educational institutions, and ambulance organisations and its clinicians should all be aware of the increased frequency of subsequent and adverse events in older adult non-conveyed patients. This study offers new insight into the association between abnormal vital signs and adverse events. A possible future clinical implication of the study findings is the implementation of age-adjusted assessments within triage systems which are specifically developed and validated for non-conveyance. Further modelling research is necessary to achieve satisfying levels of patient safety through the use of more accurate triage systems and, thereby, the creation of more favourable circumstances for ambulance clinicians to perform these most often difficult assessments. Further research is required to identify and determine relevant outcome measures for different non-conveyance populations. Moreover, there is a need for further research into the patient perspective with regard to subsequent and adverse events following non-conveyance. Despite the increasing research within the field of non-conveyance, the assessment of older adult non-conveyed patients is hindered by several aggravating circumstances and clinical challenges. Thus, this study could inspire and provide a framework which supports further studies in the field.

## Conclusion

Older adult non-conveyed patients differ significantly on several demographic and clinical characteristics compared with younger non-conveyed patients. Furthermore, the risk of subsequent and adverse events is consistently more frequent among older non-conveyed patients. Hence, indicating an ambulance service organisation system failure negatively affecting patient safety among older patients. Despite generally low mortality rates, older adult non-conveyed patients had a higher risk of dying than younger patients. The increased risk of adverse events in combination with great variety of symptom and vital sign presentations which prove difficult for dispatch operators and ambulance clinicians to identify, and assess raises questions pertinent to the patient safety of older adult non-conveyed patients.

## Supplementary Information

**Additional file 1.** Variables and cut-off points

**Additional file 2.** Unadjusted and adjusted odds-ratios for abnormal vital signs association to hospitalisation within 7 days after non-conveyance in Stockholm, Sweden, 2015. Includes both crude and adjusted odds-ratios with 95% CI, the AOR is presented in Fig. 2 (the heatmap).

## Data Availability

The data that support the findings of this study are available from the respective ambulance companies, but restrictions apply to the availability of these data, which were used under license for the current study, and so are not publicly available. Deidentified participant data are however available from the authors upon reasonable request and with permission of the respective ambulance company.

## Abbreviations

AOR: Adjusted odds-ratios
CI: Confidence interval
COR: Crude odds-ratios
ED: Emergency department
EMCC: Emergency medical communication centre
GCS: Glasgow Coma Scale
NACA: National Advisory Committee for Aeronautics
NHS: National Health Service of England
RETTS©: Rapid Emergency Triage and Treatment System
STROBE: Strengthening the Reporting of Observational Studies in Epidemiology
