# Supplementary material for: Non-conveyance of older adult patients and association with subsequent clinical and adverse events after initial assessment by ambulance clinicians: a cohort analysis
Source: BMC Emerg Med. 2021 Dec 11;21:154. doi: 10.1186/s12873-021-00548-7 (PMC8666056; doi:10.1186/s12873-021-00548-7)
Supplement: Supplementary file 1 — Additional file 1. Variables and cut-off points [file 12873_2021_548_MOESM1_ESM.docx]

| **Variable** | **Cut off point** | **Code** |
| --- | --- | --- |
| **Demographics** |  |  |
| Gender | N/A | 1. Female 2. Male |
| Age | N/A | Years |
| Priority | N/A | 1. Prio 1 2. Prio 2 3. Prio 3 |
| Month | December-February  March-May  June-August  September-November | 1. Winter 2. Spring 3. Summer 4. Autumn |
| Weekday_workday | 1. Mon-fri 2. Sat-sun | 1. Workday 2. Weekday |
| Time of day | 1. 8AM-4PM 2. 4PM-10PM 3. 10PM-8AM | 1. Day 2. Evening 3. Night |
| Geographical classification | 1. A901 2. A902 3. A903 4. A904 5. A905 6. A906 7. A907 8. A908 9. A909 10. A912 11. A913 12. A914 13. A916 14. A917 15. A918 16. A921 17. A921SUN 18. A922 19. A923 20. A924 21. A924Ö 22. A925 23. A927 24. A928 25. A929 26. A931 27. A933 28. A934 29. A935 30. A936 31. A941 32. A943 33. A943Ö 34. A951 35. A952 36. A954 37. A961 38. A965 39. A971 40. A971DAN 41. A973 42. A976 43. A976VAX 44. A976VAXÖ 45. A976Ö 46. A981 47. A981ÖST 48. A984 49. A987 50. A987SAL 51. B911 52. B911RIM 53. B911Ö 54. B912 55. B914 56. B925 57. B931 58. B931HÖL 59. B931JÄR 60. B931NKV 61. B931Ö 62. B932 63. B941 64. B941VÄS 65. B944 66. B945 67. B945Ö 68. ARN | 1. Highly urban 2. Highly urban 3. Highly urban 4. Urban 5. Urban 6. Urban 7. Highly urban 8. Urban 9. Highly urban 10. Urban 11. Highly urban 12. Urban 13. Highly urban 14. Highly urban 15. Highly urban 16. Urban 17. Urban 18. Highly urban 19. Highly urban 20. Urban 21. Urban 22. Urban 23. Highly urban 24. Urban 25. Urban 26. Urban 27. Highly urban 28. Highly urban 29. Highly urban 30. Highly urban 31. Urban 32. Urban 33. Urban 34. Urban 35. Urban 36. Urban 37. Urban 38. Urban 39. Urban 40. Urban 41. Urban 42. Urban 43. Urban 44. Urban 45. Urban 46. Urban 47. Urban 48. Urban 49. Urban 50. Urban 51. Rural 52. Rural 53. Rural 54. Rural 55. Rural 56. Rural 57. Average urban 58. Average urban 59. Average urban 60. Average urban 61. Average urban 62. Average urban 63. Urban 64. Urban 65. Urban 66. Urban 67. Urban 68. Urban |
| Geo class_code | N/A | 1. Highly urban 2. Urban 3. Average urban 4. Rural |
| **Diagnosis** |  |  |
| Prehospital diagnosis | 1. Allergi 2. Allmänt ospecificerat 3. Allmänt övrigt 4. Amputationsskada 5. Andning ospecificerat 6. Andning övrigt 7. Andningsbesvär 8. Andningsbesvär m framm. Kropp 9. Andningsbesvär m pip astma 10. Andningsstillestånd 11. Aortaaneurysm 12. Bihåleinflammation 13. Blixtnedslag 14. Blodförgiftning 15. Blödning från mun, svalg, mage-tarm 16. Blödning inre 17. Blödning under pågående graviditet 18. Blödning yttre 19. Bråck 20. Brännskada 21. Bröstkorgssmärtor 22. Buksmärta 23. Centrala bröstsmärtor – kärlkramp 24. Chock 25. Cirkulation, ospecificerat 26. Cirkulation, övrigt 27. Diabetes 28. Diabetes - högt blodsocker 29. Diabetes – lågt blodsocker 30. Diarré 31. Drog-/läkemedelsmissbruk 32. Drunkningstillbud 33. Dykskada 34. Död 35. Ej bedömning 36. Elskada 37. Embolier tromboser – artär 38. Embolier tromboser – ven 39. Epiglottit 40. Feber 41. Fraktur 42. Frätskada 43. Förgiftning med alkohol 44. Förgiftning med alkohol och läkemedel 45. Förgiftning med födoämne inkl svamp 46. Förgiftning med läkemedel 47. Förgiftning med narkotika 48. Förgiftning med tobak 49. Förgiftning ospecificerat 50. Förgiftning petroleumprod inkl etylenglykol/metanol 51. Förgiftning övrigt 52. Förkylning 53. Förlamning 54. Förlossning under pågående uppdrag 55. Förvirring/aggressivitet 56. Gallvägssjukdom 57. Gyn-blödning 58. Gynekologi/förlossning ospecificerat 59. Gynekologi/förlossning övrigt 60. Hallucinationer 61. Hemorrojder 62. Hjärnblödning/hjärninfarkt 63. Hjärnskakning 64. Hjärtinfarkt 65. Hjärtstopp - asystoli – PEA 66. Hjärtstopp - med framgångsrik HLR 67. Hjärtstopp-ventrikelflimmer 68. Hjärtsvikt 69. Huvudvärk 70. Hypertoni 71. Hypotermi kylskada 72. Hypotoni 73. Ileus 74. Illamående, kräkning 75. Infektion ospecificerat 76. Infektion övrigt (inkl smittsamma sjukdomar) 77. Inflammatoriska systemsjukdomar – RA 78. Influensa 79. Kirurgi ospecificerat 80. Kirurgi övrigt 81. Kläm-/krosskada 82. Koma/somnolent/medvetslös 83. Komplikation till värkarbete och förlossning 84. Kramper, feberkramper 85. Krisreaktion 86. Kronisk bronkit 87. Kronisk obstruktiv lungsjukdom 88. Ledsjukdom 89. Lungemfysem 90. Lunginflammation 91. Lungödem 92. Luxation 93. Maginfluensa 94. Magsår 95. Medicin ospecificerat 96. Medicin övrigt 97. Medvetanderubbning inkl svimning - andas normalt 98. Medvetandesänkning 99. Meningit 100. Missfall 101. Multipel skleros 102. Neurologi ospecificerat 103. Neurologi övrigt 104. Njursten 105. Njursvikt 106. Nyföddhetsvård 107. Nära förestående förlossning 108. Näsblödning 109. Oklara symtom 110. Omskakad 111. Ortopedi ospecificerat 112. Ortopedi övrigt 113. Oskadad 114. Pneumothorax 115. Prechock 116. Psuedokrupp 117. Psykiatri ospecificerat 118. Psykiatri övrigt 119. Psykiska symtom och sjukdomar 120. Påverkat allmäntillstånd 121. Ryggsjukdom/ischias/diskbråck 122. Rytm- eller retledningshinder 123. Rökskada 124. Sjukdom i ben- och bråskvävnad 125. Sjukdom i mjukvävnad 126. Sjukdomar i bukspottskörteln 127. Skador/olycksfall ospecificerat 128. Skador/olycksfall övrigt 129. Smärta ospecificerad 130. Social svikt 131. Subarachnoidalblödning 132. Svår skallskada 133. Sårskada 134. Transitorisk ischemisk attack 135. Tuberkolos 136. Tumör cancer 137. Urinvägs- inkl KAD-besvär 138. Utomkvedshavandeskap 139. Varicer 140. Yrsel | 1. Respiratory system 2. Other/Non-classifiable 3. Other/Non-classifiable 4. Trauma 5. Respiratory system 6. Respiratory system 7. Respiratory system 8. Respiratory system 9. Respiratory system 10. Respiratory system 11. Circulatory system 12. Infection 13. Trauma 14. Circulatory system 15. Digestive system and abdomen 16. Circulatory system 17. Obstetrics and gynaecology 18. Circulatory system 19. Digestive system and abdomen 20. Trauma 21. Circulatory system 22. Digestive system and abdomen 23. Circulatory system 24. Circulatory system 25. Circulatory system 26. Circulatory system 27. Nervous system 28. Nervous system 29. Nervous system 30. Digestive system and abdomen 31. Nervous system 32. Trauma 33. Trauma 34. Other/Non-classifiable 35. Other/Non-classifiable 36. Trauma 37. Circulatory system 38. Circulatory system 39. Respiratory system 40. Infection 41. Trauma 42. Trauma 43. Poisoning 44. Poisoning 45. Poisoning 46. Poisoning 47. Poisoning 48. Poisoning 49. Poisoning 50. Poisoning 51. Poisoning 52. Respiratory system 53. Nervous system 54. Obstetrics and gynaecology 55. Psychiatrics 56. Digestive system and abdomen 57. Obstetrics and gynaecology 58. Obstetrics and gynaecology 59. Obstetrics and gynaecology 60. Psychiatrics 61. Digestive system and abdomen 62. Nervous system 63. Trauma 64. Circulatory system 65. Circulatory system 66. Circulatory system 67. Circulatory system 68. Circulatory system 69. Nervous system 70. Circulatory system 71. Trauma 72. Circulatory system 73. Digestive system and abdomen 74. Digestive system and abdomen 75. Infection 76. infection 77. Medicine 78. Infection 79. Trauma 80. Trauma 81. Trauma 82. Nervous system 83. Obstetrics and gynaecology 84. Nervous system 85. Psychiatrics 86. Respiratory system 87. Respiratory system 88. Medicine 89. Respiratory system 90. Respiratory system 91. Respiratory system 92. Trauma 93. Digestive system and abdomen 94. Digestive system and abdomen 95. Medicine 96. Medicine 97. Nervous system 98. Nervous system 99. Nervous system 100. Obstetrics and gynaecology 101. Medicine 102. Nervous system 103. Nervous system 104. Digestive system and abdomen 105. Digestive system and abdomen 106. Other/Non-classifiable 107. Obstetrics and gynaecology 108. Other/Non-classifiable 109. Other/Non-classifiable 110. Other/Non-classifiable 111. Trauma 112. Trauma 113. Other/Non-classifiable 114. Respiratory system 115. Circulatory system 116. Respiratory system 117. Psychiatrics 118. Psychiatrics 119. Psychiatrics 120. Other/Non-classifiable 121. Other/Non-classifiable 122. Circulatory system 123. Trauma 124. Other/Non-classifiable 125. Other/Non-classifiable 126. Digestive system and abdomen 127. Trauma 128. Trauma 129. Other/Non-classifiable 130. Other/Non-classifiable 131. Nervous system 132. Trauma 133. Trauma 134. Nervous system 135. Infection 136. Other/Non-classifiable 137. Digestive system and abdomen 138. Obstetrics and gynaecology 139. Other/Non-classifiable 140. Nervous system |
| Initial diagnosis | N/A | \| 1. Circulatory system \| \| --- \| \| 1. Nervous system \| \| 1. Digestive system and abdomen \| \| 1. Infection \| \| 1. Medicine \| \| 1. Obstetrics and gynaecology \| \| 1. Other/Non-classifiable \| \| 1. Psychiatrics \| \| 1. Respiratory system \| \| 1. Trauma \| |
| **Treatment and actions** |  |  |
| Drugs | N/A | 1. No drugs 2. Drugs |
| Actions | N/A | 1. No actions 2. Actions |
|  |  |  |
| **Vital functions and observational scales** |  |  |
| Respiratory rate (/min) | Adults   1. 8-25/min 2. <8/min 3. >25/min   Children 0-1 year   1. 25-45/min 2. <25/min 3. >45/min   Children 2-3 year   1. 20-35/min 2. <20/min 3. >35/min   Children 3-5 year   1. 16-24/min 2. <16/min 3. >24/min   Children 6-11 year   1. 14-20/min 2. <14/min 3. >20/min   Children 12-18 year   1. 8-25/min 2. <8/min 3. >25/min | 1. Normal 2. Abnormal (too low) 3. Abnormal (too high) 4. Normal 5. Abnormal (too low) 6. Abnormal (too high) 7. Normal 8. Abnormal (too low) 9. Abnormal (too high) 10. Normal 11. Abnormal (too low) 12. Abnormal (too high) 13. Normal 14. Abnormal (too low) 15. Abnormal (too high) 16. Normal 17. Abnormal (too low) 18. Abnormal (too high) |
| Oxygen saturation (%) | 1. 95-100% 2. <95% | 1. Normal 2. Abnormal (too low) |
| Heart rate (/min) | Adults   1. 50-110/min 2. <50/min 3. >110/min   Children 0-1 year   1. 80-140/min 2. <80/min 3. >140/min   Children 2-3 year   1. 75-130/min 2. <75/min 3. >130/min   Children 3-5 year   1. 70-115/min 2. <70/min 3. >115/min   Children 6-11 year   1. 65-110/min 2. <65/min 3. >110/min   Children 12-18 year   1. 55-110/min 2. <55/min 3. >110/min | 1. Normal 2. Abnormal (too low) 3. Abnormal (too high) 4. Normal 5. Abnormal (too low) 6. Abnormal (too high) 7. Normal 8. Abnormal (too low) 9. Abnormal (too high) 10. Normal 11. Abnormal (too low) 12. Abnormal (too high) 13. Normal 14. Abnormal (too low) 15. Abnormal (too high) 16. Normal 17. Abnormal (too low) 18. Abnormal (too high) |
| Systolic blood pressure (mmHg) | Adults   1. <90 mmHg 2. 90-160 mmHg 3. 160 mmHg | 1. Abnormal (too low) 2. Normal 3. Abnormal (too high) |
| Temperature (℃) | 1. <35 ℃ 2. 35-38,5 ℃ 3. >38,5 ℃ | 1. Abnormal (too low) 2. Normal 3. Abnormal (too high) |
| Blood glucose level (mmol/L) | 1. <3,5 mmol/L 2. 3,5-14 mmol/L 3. 14 mmol/L | 1. Abnormal (too low) 2. Normal 3. Abnormal (too high) |
| Nervous system | \| One of the following: Disoriented \| \| --- \| \| Incomprehensible sounds \| \| Incoherent \| \| Unconscious \| \| No reaction   1. Oriented \| | 1. Abnormal 2. Normal |
| Glasgow Coma Scale (EMV) | 1. EMV < 15 2. EMV > 15 | 1. Abnormal 2. Normal |
| **Conveyance** | N/A | 1. Conveyed 2. Non-conveyed |
| **NACA** | N/A | 1. NACA 0 2. NACA 1 3. NACA 2 4. NACA 3 5. NACA 4 6. NACA 5 7. NACA 6 8. N/A 9. “Vet ej” |
|  |  |  |
|  |  |  |
