# Supplementary material for: Non-conveyance of older adult patients and association with subsequent clinical and adverse events after initial assessment by ambulance clinicians: a cohort analysis
Source: BMC Emerg Med. 2021 Dec 11;21:154. doi: 10.1186/s12873-021-00548-7 (PMC8666056; doi:10.1186/s12873-021-00548-7)
Supplement: Supplementary file 2 — Additional file 2. Unadjusted and adjusted odds-ratios for abnormal vital signs association to hospitalisation within 7 days after non-conveyance in Stockholm, Sweden, 2015. Includes both crude and adjusted odds-ratios with 95% CI, the AOR is presented in Fig. 2 (the heatmap). [file 12873_2021_548_MOESM2_ESM.docx]

| **Abnormal vital signs** | **18-64** | | **65-74** | | **75-84** | | **85-94** | | **95-109** | |
| --- | --- | --- | --- | --- | --- | --- | --- | --- | --- | --- |
|  | UOR (95% CI) | AOR (95% CI) | UOR (95% CI) | AOR (95% CI) | UOR (95% CI) | AOR (95% CI) | UOR (95% CI) | AOR (95% CI) | UOR (95% CI) | AOR (95% CI) |
| Respiratory rate high (>25/min) | 1 | 1 | 0.53 (0.18-1.55) | 0.67 (0.20-2.16) | 0.44 (0.17-1.12) | 0.50 (0.17-1.46) | 1.44 (0.80-2.59) | 1.71 (0.86-3.38) | 0.20 (0.05-0.81) | 0.24 (0.06-1.02) |
| Oxygen saturation abnormal (<95%) | 1 | 1 | 6.74 (4.09-11.12) | 5.15 (2.87-9.23) | 7.86 (5.18-11.94) | 7.00 (4.36-11.25) | 4.26 (2.97-6.11) | 3.30 (2.17-5.03) | 4.24 (2.92-6.15) | 3.30 (2.16-5.04) |
| Heart rate low (<50/min) | 1 | 1 | N/A | N/A | N/A | N/A | 10.12 (2.47-41.54) | 9.80 (2.24-42.81) | N/A | N/A |
| Heart rate high (>110/min) | 1 | 1 | 0.25 (0.11-0.60) | 0.19 (0.06-0.54) | 0.43 (0.24-0.79) | 0.49 (0.26-0.93) | 0.51 (0.29-0.91) | 0.51 (0.27-0.97) | 0.48 (0.25-0.90) | 0.50 (0.25-1.01) |
| Systolic blood pressure low (<90 mmHg) | 1 | 1 | N/A | N/A | N/A | N/A | 4.80 (1.51-15.29) | 4.46 (1.17-17.01) | 2.29 (0.51-10.33) | 1.58 (0.31-7.92) |
| Systolic blood pressure high (>160 mmHg) | 1 | 1 | 3.26 (1.81-5.89) | 3.20 (1.75-5.85) | 3.59 (2.22-5.82) | 3.72 (2.27-6.11) | 2.21 (1.46-3.34) | 2.18 (1.40-3.39) | 2.29 (1.46-3.59) | 1.92 (1.20-3.08) |
| Body temperature low (<35 ℃) | 1 | 1 | 9.01 (2.34-34.72) | 11.42 (2.56-51.03) | 2.52 (0.32-20.08) | 2.93 (0.35-24.76) | N/A | N/A | 1.41 (0.17-11.33) | 2.16 (0.25-19.07) |
| Body temperature high (>38.5 ℃) | 1 | 1 | 1.16 (0.35-3.84) | 0.92 (0.27-3.15) | 3.09 (1.60-5.95) | 2.63 (1.26-5.50) | 1.37 (0.59-3.20) | 1.29 (0.50-3.34) | 1.17 (0.45-2.99) | 1.06 (0.40-2.83) |
| Blood glucose level low (<3.5 mmol/L) | 1 | 1 | 0.15 (0.02-1.16) | 0.27 (0.03-2.32) | 0.55 (0.15-1.94) | 0.78 (0.21-2.84) | N/A | N/A | N/A | N/A |
| Blood glucose level high (>14 mmol/L) | 1 | 1 | 0.93 (0.12-7.06) | 1.23 (0.16-9.53) | 4.01 (1.59-10.12) | 2.57 (0.74-8.91) | 5.36 (2.76-10.40) | 4.31 (2.06-9.01) | 2.14 (0.75-6.13) | 2.42 (0.82-7.12) |
| Glascow Coma Scale abnormal (<15) | 1 | 1 | 2.82 (1.61-4.94) | 1.58 (0.75-3.34) | 1.54 (0.89-2.67) | 2.09 (1.11-3.97) | 1.52 (0.93-2.48) | 1.89 (1.10-3.24) | 2.23 (1.42-3.49) | 2.79 (1.72-4.52) |

Younger non-conveyed patients, 18-64 years, as reference group.

'Respiratory rate low' excluded because of no events. N/A= No cases
All included analyses were adjusted for gender, dispatch priority, time of day, geographical location, NACA-score, prehospital initial assessment code and triage level.

COR= Crude odds-ratio; AOR= Adjusted odds-ratio.
